# Theoretical and Experimental Studies on AC Electric Field Induced Droplet Deformation in a Microchannel

**DOI:** 10.3390/mi17091008

**Published:** 2026-08-26

**Authors:** Zuxuan Shen, Jingwen Yang, Zifeng Tan, Jiapeng Zhao, Caichan Li, Yage Zhang, Xinyu Liu

**Affiliations:** 1 School of Biomedical Engineering, Shenzhen University Medical School, Shenzhen University, Shenzhen 518060, China; 2 Guangdong Key Laboratory of Biomedical Measurements and Ultrasound Imaging, School of Biomedical Engineering, Shenzhen University Medical School, Shenzhen University, Shenzhen 518060, China

**Keywords:** microfluidics, electrohydrodynamics, droplet deformation, AC electric fields

## Abstract

We present both theoretical and experimental studies on the deformation of aqueous droplets in an alternating-current (AC) electric field. Droplets are first generated using a microfluidic T-junction and are then deformed downstream using a set of coplanar microelectrodes. We investigated how the electric field strength, AC frequencies, conductivities of the aqueous fluids and interfacial tension affect the deformation of droplets in a microchannel. The experimental results are well explained by the electrohydrodynamic (EHD) theory for almost all system parameters explored in this study. Only at very high electric field strengths do the experimental results deviate slightly from the EHD theory. The observed experimental trends can be explained by a series of scaling analyses that provide insights into the interaction of the different physical properties with the electric field. We expect that the present work will provide a better understanding of electrically mediated deformation of droplets in a microchannel.

## 1. Introduction

Droplet deformation induced by an electric field is a typical electrohydrodynamic (EHD) problem. This electrically mediated deformation is often governed by classical electrical and hydrodynamic laws at the macroscale. For example, in Taylor’s leaky dielectric model, a small amount of electrical conductivity is sufficient to allow free charges to reach the droplet–medium interface [1]. The proposed model explains the behavior of droplets deformed by a steady electric field. The underlying assumptions in the model include weak deformation, leaky dielectric media, a quasi-static electric field, and no charge convection inside the droplet. As a result, the theoretical deformation of a static droplet was derived from the stress distribution resulting from the electric field [1]. Torza et al. later extended the theory to droplets in AC electric fields [2]. The experimental data were found to be qualitatively consistent with the EHD theory. Visika and Saville studied the deformation of static droplets in both direct-current (DC) and alternating-current (AC) electric fields. The experimental data were also found to be quantitatively consistent with the existing EHD theory [3]. In the case of Newtonian droplets, Ha and Yang observed that the responses of highly conducting droplets are well described by the electrohydrostatic theory [4].

In the above pioneering works, most studies were conducted at the macroscale. The previous works also focused mainly on static droplets deformed by an electric field. It is interesting that few studies have focused on both theoretical and experimental studies of droplet deformation in a microchannel, despite the large number of applications developed using microfluidic devices. These microscale applications include common droplet manipulation tasks such as binning, splitting, sorting and generation that involve the deformation of interfaces [5,6,7,8,9]. In most of these cases, the use of an AC electric field is often preferred due to a variety of factors. For example, the use of AC electric fields is found to be less harmful to living cells. This is because the voltage across the capacitive membrane of the cell is relatively lower than the DC case [10]. In other cases, AC electric fields avoid ion screening, which may result in unstable transient behaviors [10,11,12,13,14]. Nevertheless, the use of AC electric fields has been widely adopted and preferred in most droplet-based microfluidic applications. Hence, a better understanding of this topic will serve to elucidate and bridge the gap between the previous literature and the current experimental works in microchannels.

In a previous work, we briefly demonstrated that aqueous droplets in a microchannel can be deformed using an AC electric field [15]. Non-contact coplanar electrodes, which are insulated by Poly(dimethylsiloxane) (PDMS) walls, help to prevent electrolysis, which commonly occurs when fluids are in contact with the electrodes. In this work, we systematically investigate various parameters such as the electric field strength, AC frequencies, conductivities of the aqueous fluids and interfacial tensions between the fluids. The electric field strength was limited to $E=2.65\times 10^{6}$ (V/m), well below the dielectric breakdown strength of PDMS [16]. In this range, we also maintained small deformation of droplets that can be described by the leaky dielectric model [1,4]. It is worth noting that electric fields in microfluidic devices with coplanar electrodes can also produce dielectrophoretic (DEP) forces [17,18,19], though the DEP forces primarily govern droplet motion and are fundamentally different from the EHD deformation studied in the present work. We also observed experimentally that if the electric field strength is too high, unstable droplet deformation or droplet breakup will occur, leading to various instabilities [20]. We varied the AC frequency from f=10 kHz to f=70 kHz as this range is commonly used in the above droplet manipulation tasks [7,8,11]. In the previous work, we also observed that unstable droplet deformation occurs when f is below 10 kHz [15]. The electrical conductivities of the aqueous fluids were varied by seven orders of magnitudes. This leads to a variation in the conductivity ratio R to be between the orders of $10^{7}$ to $10^{13}$. The interfacial tensions between the fluids are varied between 56 to 29 mN/m by mixing water with pure ethanol solutions. Our scaling analyses show that the proposed modified electric capillary number $Ca_{E}^{\prime }$ can be used to account for the competition between the electric field strength and interfacial tensions.

## 2. Theoretical Background

When a steady electric field is applied to a static droplet suspended in another fluid, the droplet will deform into either a prolate or oblate shape. This deformation is relative to the direction of the electric field. Taylor suggested that the fluids should be treated as leaky dielectrics with finite electrical conductivity. The conduction process would allow free charges to accumulate on the interface between the droplet and the surrounding fluid. As a result, the action of the electric field on the surface free charge produces tangential stresses that set the viscous flow into motion [1]. Based on the stress distribution, Taylor obtained an analytical solution for the droplet deformation D:

(1) $$D=\frac{9}{16}\frac{a\epsilon_{0}\epsilon_{1}E^{2}}{\gamma }\phi$$

where D=(L−B)/(L+B), with L and B denoting droplet dimensions parallel and perpendicular to the direction of the electric field, respectively. Here, a is the radius of the undeformed droplet, $\epsilon_{0}$ is the vacuum dielectric constant, $\epsilon_{1}$ is the relative permittivity of surrounding fluid, γ is the surface tension, and ϕ is the discriminant function:

(2) $$\phi =S\left(R^{2}+1\right)-2+3\left(SR-1\right)\frac{2M+3}{5M+5}$$

where R is the conductivity ratio between the droplet and the medium ($R=\sigma_{2}/\sigma_{1}$), S is the permittivity ratio between the medium and the droplet ($S=\epsilon_{1}/\epsilon_{2}$), and M is the viscosity ratio between the medium and the droplet ($M=\mu_{1}/\mu_{2}$). The subscripts ‘1’ and ‘2’ correspond to the oil phase and the aqueous phase, respectively. In our case, the aqueous phase is the droplets of various salt solutions. When ϕ>0, the droplet undergoes prolate deformation, while for ϕ<0, the droplet undergoes oblate deformation.

Taylor’s leaky dielectric model successfully predicts the shape of the deformed droplet as well as the recirculation flow inside the droplet induced by the electric field. Torza et al. generalized Taylor’s theory for the case of an AC field, where the electric field varies as $E=E_{\infty }\cos\omega t$, and ω=2πf is the angular frequency of the electric field [2]. The model indicated that droplet deformation has both a steady component $D_{S}$ and oscillatory component $D_{T}$, where $D=D_{S}+D_{T}$ and

(3) $$D_{S}=\frac{9}{16}\frac{a\epsilon_{1}\epsilon_{0}E^{2}}{\gamma }\phi_{S}$$

E is the electric field strength. We define E=V/d, where V is the root mean square voltage and d is the distance between two electrodes. *E* is defined as a characteristic effective field strength for the droplet in the central observation region, where the field gradient is minimal, and the projected droplet diameter (~50–60 μm) is much smaller than the electrode gap (170 μm). No measurable dielectrophoretic translation or asymmetric deformation is observed in this region, supporting the local-uniform-field approximation. Equation (3) has the same form as Equation (1), only the discriminant function is different:

(4) $$\begin{array}{cc} \phi_{S}= & 1-\\ & \frac{S^{2}R\left(11+14M\right)+15S^{2}\left(1+M\right)+S\left(19+16M\right)+15R^{2}S\tau^{2}\omega^{2}\left(M+1\right)\left(S+2\right)}{5\left(M+1\right)\left[S^{2}{\left(2+R\right)}^{2}+R^{2}\tau^{2}\omega^{2}{\left(1+2S\right)}^{2}\right]} \end{array}$$

where $\tau =(\epsilon_{0}\epsilon_{1})/\sigma_{2}$ is the hybrid electrical relaxation time. It should be noted that when ω=0, Equation (4) reduces to Equation (2). The oscillatory component $D_{T}$ varies with time at a frequency of 2ω. For a given droplet–medium system (fixed interfacial tensions), when $\omega \mu_{1}\to \infty$, we have $(D_{T}/D_{S})\to 0$, and droplet deformation has no response to the oscillatory component of the electrical stresses. This is the case in our experiments. When the magnitude of $\omega \mu_{1}$ is small, the droplet will experience slow periodic deformation, which differs from the behavior reported in previous studies [3,21,22,23].

## 3. Materials and Methods

**Figure 1:**
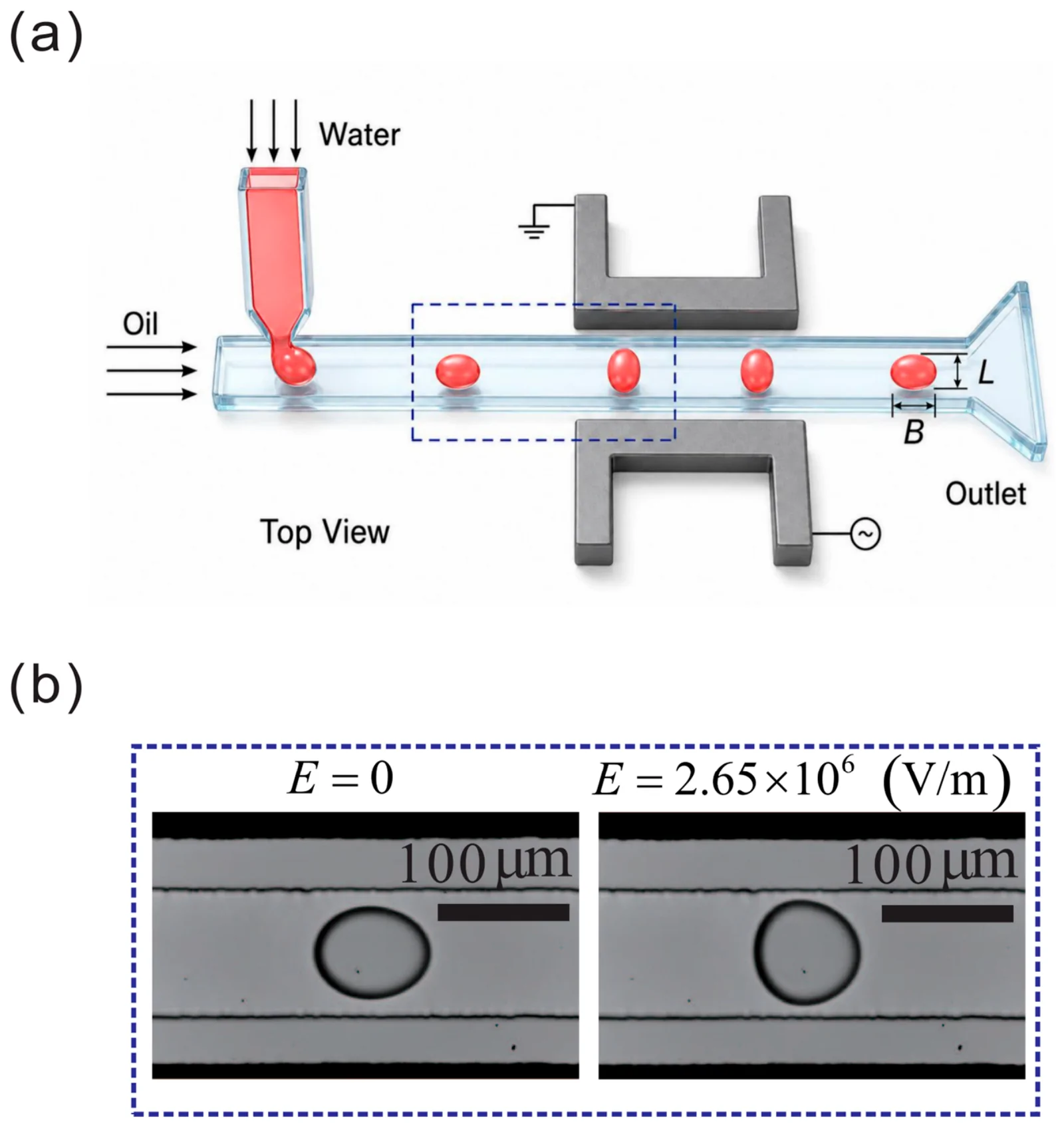
(**a**) Schematic sketch of the microchannel. (**b**) Droplet with and without electric field. The scale bar indicates 100 μm. The dispersed phase fluid is represented in red. The dotted box illustrates the deformation of the droplets before and after entering the electric field region.

Figure 1a shows the schematic sketch of the microfluidic device. The device was fabricated with polydimethylsiloxane (PDMS) using standard photolithography and soft lithography. The PDMS microchannels were bonded to an ITO-coated glass slide. This ITO-coated glass slide was then grounded to prevent floating voltages [11]. The width and height of the microchannel are 100 μm and 30 μm, respectively. Droplets are formed using an orifice of 20 μm. The electrodes are manufactured using microsolidics [24] and placed about 3000 μm away from the dispersed phase channel. We anticipate that the electric field does not affect the droplet formation process and no significant size variations are observed during the experiments [15]. The distance between the two electrodes is about 170 μm. We used mineral oil ($\epsilon_{c}=2.3$, M5904, Sigma-Aldrich, St. Louis, MO, USA) as the continuous phase and DI water ($\epsilon_{d}=80$), sodium chloride solutions and ethanol solutions of different concentrations as the dispersed phase. DI water I was obtained from the Milli-Q Direct 8 system (Merck Millipore, Burlington, MA, USA) and DI water II was obtained from a centralized distillation tap. The change in electrical permittivity of the dispersed phase due to the addition of sodium chloride is calculated based on the model proposed by [25]. The conductivities were measured by a conductivity meter (HI5521-02, Hanna Instruments, Woonsocket, RI, USA) and the interfacial tensions were measured using the pendant-drop method (PAT-1, Sinterface, Berlin, Germany). All devices were treated with Aquapel (PPG Industries, Pittsburgh, PA, USA) to render the surfaces hydrophobic.

The volumetric flow rates were controlled by two syringe pumps (Cetoni, Korbussen, Germany). The flow rates for the continuous phase (CP) and dispersed phase (DP) were fixed at 600 μL/h and 75 μL/h, respectively. These flow rates were chosen such that the droplets would not be confined by the side channel walls even at maximum deformation. The corresponding flow velocity of the continuous and dispersed phases was 5.56 cm/s and 0.69 cm/s, respectively. However, we note that the droplets were confined in the vertical direction by the top and bottom channel walls due to the channel height. Sinusoidal voltages at frequencies from 10 kHz to 70 kHz (33210A, Agilent, Santa Clara, CA, USA) and amplified from 0 to ~450 V (623B, Trek, Lockport, NY, USA) were applied on the electrodes. Voltages are in root mean square (RMS) unless otherwise mentioned. For each time–frequency variation, the output voltage was monitored until the prescribed *V_RMS_* was obtained to isolate the effect of frequency only. Droplets were imaged by an inverted microscope (Ti-E, Nikon, Tokyo, Japan) with a high-speed camera (Miro3, Phantom, Vision Research, Wayne, NJ, USA, at 2000 frames/s). The droplet size and deformation were measured automatically from images of at least 20 different droplets by OpenCV-based Automated Droplet Measurement (ADM) software [26]. Table 1 shows that in all cases in our experiments, SR≫1 and the droplets were always stretched in the direction of the electric field [1] (Figure 1b).

## 4. Results and Discussion

### 4.1. The Effects of AC Electric Field Strength and Frequency on Droplet Deformation

We first investigated the effects of electric field strength (E) and frequency (f) on the deformation of droplets. Droplet radius (a) is calculated as $a=\sqrt{A/\pi }$, where A is the area of the droplet. In our experimental system, f is on the order of $O(10^{4})$ Hz. Using the EHD theory, we estimate that ${\left(D_{T}/D_{S}\right)}_{max}≲0.1$; thus, $D\approx D_{S}$. This approximation is also verified by the fact that only steady droplet deformation was observed in our experiments.

**Figure 2:**
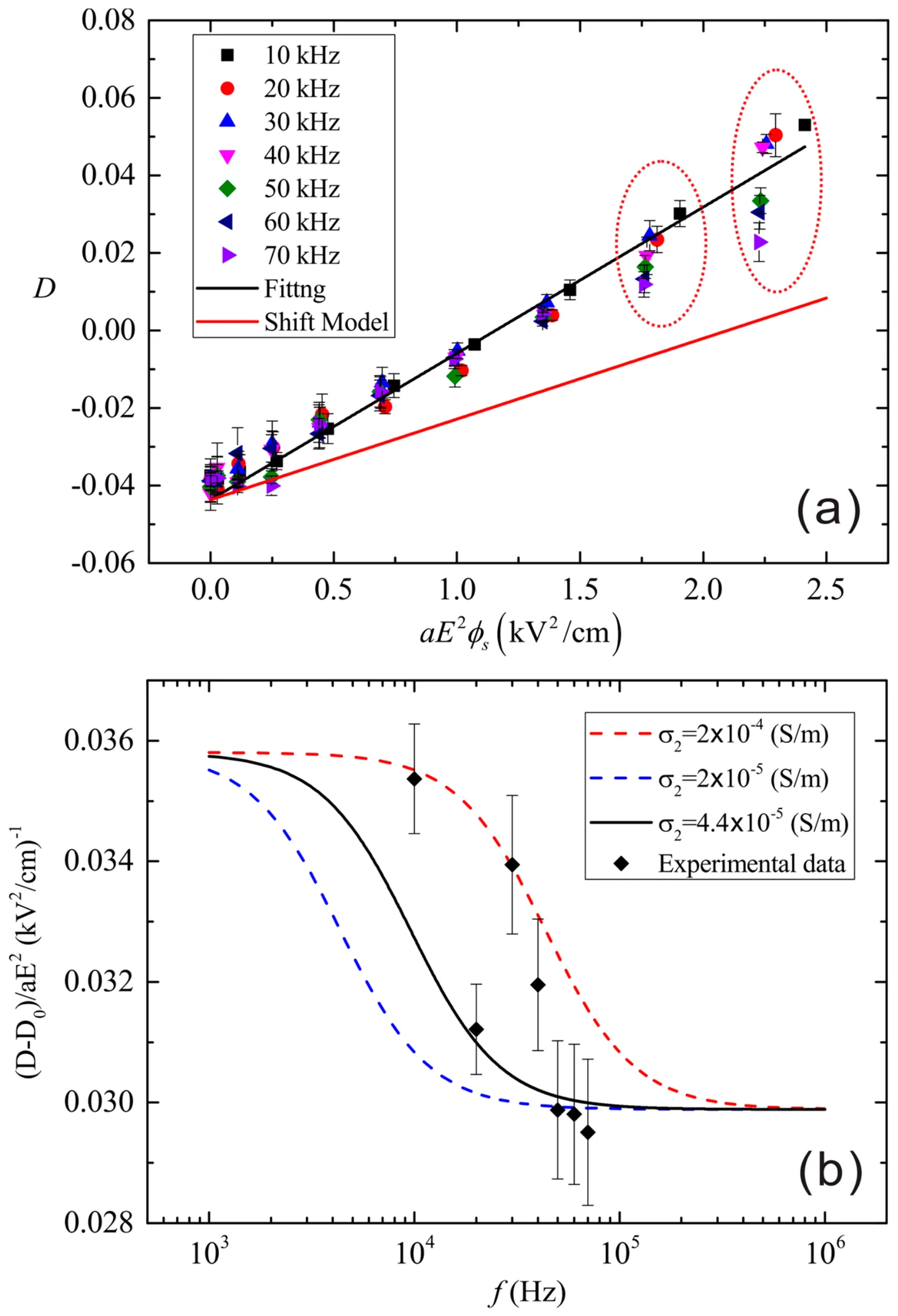
(**a**) Droplet (DI water I) deformation D as a function of $aE^{2}\phi_{S}$ for AC frequencies between 10 kHz and 70 kHz. The black solid line is a linear fitting of the experimental results with Equation (5). The red solid line is calculated from Equation (3). (**b**) Normalized droplet deformation as a function of AC frequencies. The lines are plotted based on Equation (6). The black solid line is obtained using the measured electrical conductivity of DI water I. The dashed lines are based on the theoretical electrical conductivities in order to fit the experimental data points.

Figure 2a shows the droplet deformation D as a function of $aE^{2}\phi_{S}$ for frequency f ranging from 10 kHz to 70 kHz. D has an initial negative value when V=0 and increases almost linearly with the increasing $aE^{2}\phi_{S}$. The initial negative value is due to the vertical confinement of the top and bottom channel walls and the hydrodynamic forces induced by the flow. As a result, the droplets resemble a “bullet” shape with a higher radius of curvature in front and lower curvature at the back (Figure 1b). The observed linear increase is consistent with the EHD theory (see Equation (3)). Our scaling analysis shows that the experimental data can be fitted by

(5) $$D=k_{e}\cdot aE^{2}\phi_{S}+D_{0}$$

which is represented by the black line in Figure 2a. $k_{e}$ is the fitting slope with the dimension of $m\cdot V^{-2}$, and $D_{0}$ is the fitted intercept. In this case, we treat $D_{0}$ as the initial droplet deformation due to the confinement and hydrodynamic forces. When we take $D_{0}$ as the initial droplet deformation, the prediction based on Equation (3) can be represented by the red solid line in Figure 2a. The corresponding slope is $k_{th}=(9/16)(\epsilon_{0}\epsilon_{1}/\gamma )$. We found that the ratio of the two slopes $k_{e}/k_{th}=1.79$ is comparable and is an acceptable estimate. According to the existing literature, the slopes of [3] and that of [2] are within 1.0∼1.4 and 2.1∼3.8, respectively (Table 2). Hence, it is reasonable to conclude that the leaky dielectric model can be used in the case of droplet deformation in a microchannel.

Figure 2a shows that when the E is large ($aE^{2}\phi_{S}>1.75\times 10^{8}$ $V^{2}/m$), the experimental data points are scattered more than those at low E ($aE^{2}\phi_{S}<1.75\times 10^{8}$ $V^{2}/m$). D is smaller when f is high, as shown in Figure 2a. We attribute this to the frequency dependence of the electrical conductivity, i.e., the electrical conductivity of the aqueous solution decreases with increasing AC frequencies [27]. This inevitably leads to a decreasing $\phi_{S}$ in Equation (4), which in turn decreases $D_{s}$ in Equation (3). In Equation (5), $\phi_{S}$ is the only parameter that relates to f. In order to explore the role of AC frequencies in droplet deformation, we rewrite Equation (5) as

(6) $$\frac{D-D_{0}}{aE^{2}}=k_{e}\cdot \phi_{S}\left(f\right)$$

and then plot the normalized droplet deformation $\frac{D-D_{0}}{aE^{2}}$ as a function of the f (Figure 2b). The diamond symbols are experimental data, and the black solid line is the theoretical value of $k_{e}\cdot \phi_{S}\left(f\right)$ at different AC frequencies. The normalized droplet deformation decreases with increasing AC frequencies. The experimental data agree well with theoretical predictions where the maximum deviation is less than 10%.

Figure 2b also shows that the normalized droplet deformation is very sensitive to the electrical conductivity, except for the solid black line, which is based on the measured electrical conductivity ($\sigma_{2}=4.4\times 10^{-5}$ S/m). The blue dashed line represents a lower conductivity ($\sigma_{2}=2\times 10^{-5}$ S/m), and the red dashed line represents a higher conductivity ($\sigma_{2}=2\times 10^{-4}$ S/m). These comparisons indicate that at low AC frequency (~10 kHz), the experimental data are close to the theoretical values of higher droplet conductivity, while at high AC frequency (>50 kHz), the experimental data are close to the theoretical values of lower conductivity. The electrical conductivity decreases with increasing frequency f. We would like to highlight that this observation is consistent with the Drude model, which focuses on the transport properties of charges and electrons in materials [27].

### 4.2. The Effect of Droplet Electrical Conductivity on Droplet Deformation

We investigated the effects of electrical conductivities on droplet deformation using sodium chloride solutions. To the best of our knowledge, few studies have examined the deformation of highly conducting aqueous droplets in a microchannel using an AC electric field. In the case of droplet deformation in a microchannel, most previous studies focused on experimental applications where different droplet tasks are explored and demonstrated. We used aqueous sodium chloride solutions of different concentrations, with conductivities ranging across six orders of magnitude and R ranging from $10^{7}$ to $10^{13}$ (Table 1). The measured interfacial tensions do not vary significantly with the addition of a small amount of sodium chloride into DI water.

**Figure 3:**
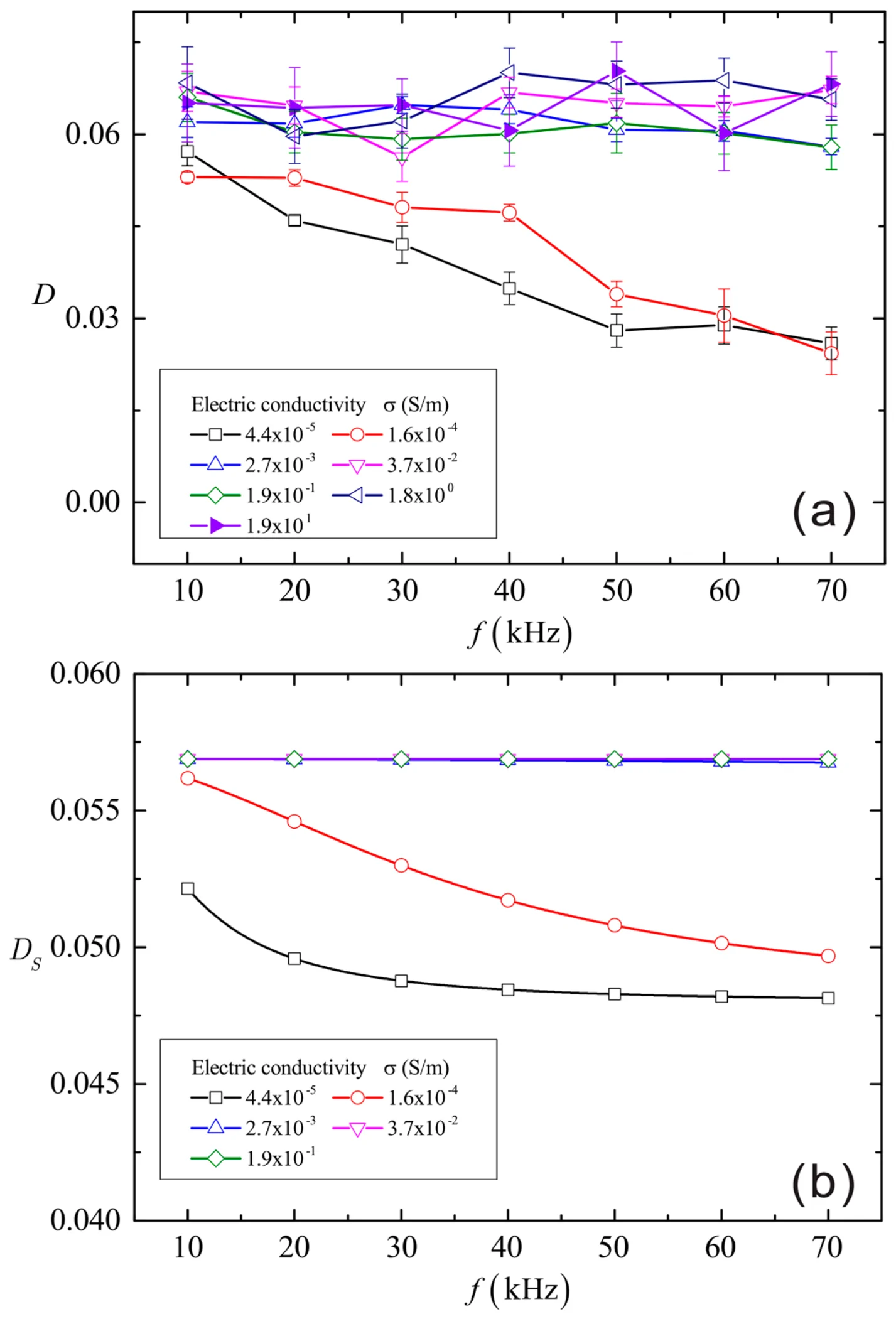
(**a**) Droplet deformation D as a function of AC frequencies for electrical conductivities ranging from $4.4\times 10^{-5}$ S/m to $1.9\times 10^{1}$ S/m. The electric field strength is kept at $E^{2}=7.0\times 10^{12}(V/m{)}^{2}$. (**b**) $D_{S}$ as a function of AC frequencies. The five selected conductivities and the colors of the curves for different conductivities remain the same as those in (**a**).

Experimental results show that for droplets with higher electrical conductivity, D increases almost linearly with increasing $E^{2}$. This observation is similar to the case of DI water. Figure 3a shows the frequency dependence of the droplet deformation for different electrical conductivities. For the case of DI water I and II, D decreases with increasing frequencies. However, for the aqueous sodium chloride solutions, D shows negligible dependence on f. Across the entire frequency range explored in this study, aqueous sodium chloride solutions were observed to deform more than the case of DI water. This is in accordance with the EHD theory, where the electrical conductivity dependence of the droplet deformation is given in Equations (3) and (4). Figure 3b depicts the calculated $D_{S}$ as a function of f from 10 kHz to 70 kHz. We see that $D_{S}$ has similar trends as the experimental data shown in Figure 3a. Hence, it is reasonable to conclude that the experimental results are qualitatively consistent with the EHD theory. However, for the cases of lower electrical conductivities ($\sigma =4.4\times 10^{-5}\;\left(S/m\right)$ and $\sigma =1.6\times 10^{-4}\;\left(S/m\right)$), deviations from the EHD theory can be observed when the frequency f increases from 10 to 70 kHz. This observation confirms our conclusion regarding the f dependence of electrical conductivity: if we increase the AC frequencies, the electrical conductivity of aqueous solution will decrease. This will in turn decrease the deformation of the droplets. We emphasize that we focus here mainly on the droplet deformation in the framework of the leaky dielectric model and neglected the electrokinetic effects. As pointed out in previous studies [28,29], if electrokinetic effects such as the charge transportation and diffused charge layers are taken into account, the results will be similar to that of the leaky dielectric model. Comprehensive studies on droplet deformation from electrokinetic aspects can be found in Pillai et al.’s recent work [30,31].

### 4.3. The Effect of Interfacial Tensions on Droplet Deformation

We then investigated the effects of interfacial tensions on droplet deformation under various electric field strengths. This was achieved using aqueous ethanol solutions with concentrations of 0 wt%, 5 wt%, 10 wt% and 15 wt%. The respective surface tensions γ are 56, 44, 35 and 29 mN/m. We would like to highlight that experimentally, we observed that uniform droplets cannot be generated at higher concentration of ethanol mixtures. We postulate that this may be due to wettability issues with the channel walls, which were hydrophobic after the surface treatment. The measured electrical conductivities of these aqueous solutions were similar to that of DI water. The frequency of the sinusoidal voltage signal was fixed at 10 kHz to maximize the electrical stresses on droplets. The dispersed- and continuous-phase flow rates were fixed as mentioned previously. In order to quantify the role of interfacial tensions under the influence of an AC electric field, we used the electric capillary number, which is defined as $Ca_{E}=(a\epsilon_{0}\epsilon_{1}E^{2})/\gamma$. This dimensionless number measures the competition between the electric field strength and interfacial tension. Here, $\phi_{S}$ is calculated to be around 0.92 for f = 10 kHz and $\sigma_{2}∼O\left(10^{-5}\right)$ S/m. As a result, $D=0.52Ca_{E}$, which shows that the electric field strength and the interfacial tensions are within the same order of magnitude.

**Figure 4:**
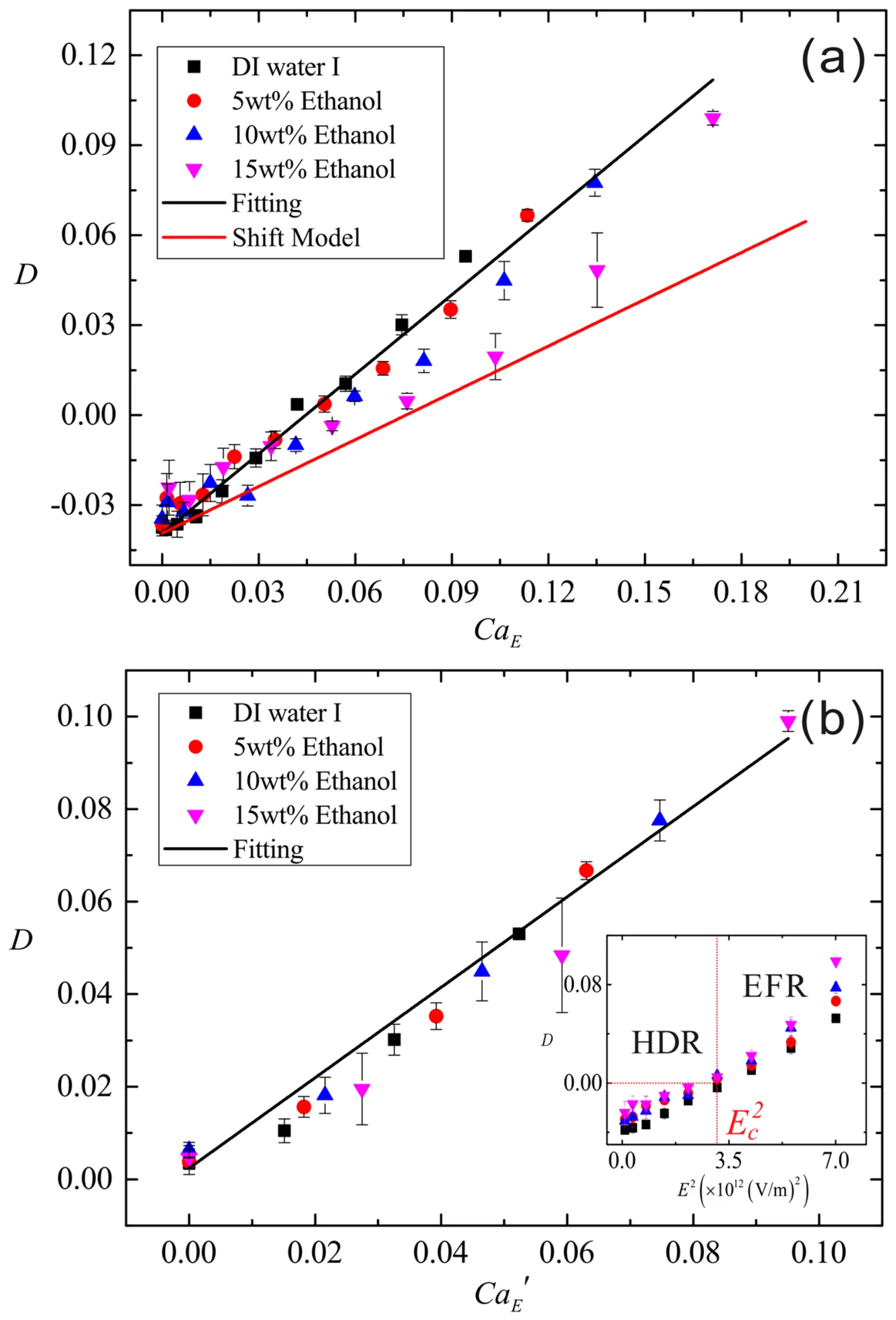
(**a**) Deformation D as a function of electric capillary number $Ca_{E}$. The symbols show four different aqueous mixtures with different interfacial tension values. The AC frequency is fixed at f = 10 kHz. The black solid line is a linear fitting of the experimental data. The red solid line is the modified theoretical prediction with an offset. This is done in order to share the same origin with the black line. (**b**) Deformation D as a function of the modified electric capillary number $Ca_{E}^{\prime }$. The data points were extracted from the regime where the electrical stress dominates ($E^{2}>{E_{c}}^{2}$) over the hydrodynamic forces. The black line is the linear fitting of the experimental data. Here, $D\approx 0.98Ca_{E}^{\prime }$. The inset in (**b**) shows deformation D as a function of $E^{2}$. HDR refers to the hydrodynamic-force-dominated regime and EFR refers to the electric-force-dominated regime.

Figure 4a shows the experimental droplet deformation as a function of the electric capillary number. Droplet deformation generally increases with increasing $Ca_{E}$. The black solid line is the linear fitting of the experimental data. The red solid line is the theoretical prediction with a slope of 0.52. We modified the prediction with an offset such that it shares the same origin with the black line. The ratio of the two slopes is 1.69, which is consistent with the results in Section 4.1. In Figure 4a, the experimental data are scattered when $Ca_{E}$ increases for surface tensions of 35 and 29 (mN/m) (blue and purple triangle symbols). In order to take into account this difference and to understand the effect of interfacial tensions with the electric field strength, we propose a modified electric capillary number $Ca_{E}^{\prime }=a\epsilon_{0}\epsilon_{1}\left(E^{2}-E_{c}^{2}\right)/\gamma$, where $E_{c}$ represents the critical electric field strength where the electric forces are balanced by hydrodynamic forces and *a* represents the different droplet radius. At the critical electric field strength $E_{c}$, we observed that the droplets are almost circular in shape rather than prolate. Figure 4b shows droplet deformation as a function of $Ca_{E}^{\prime }$. The modified scaling shows a linear relationship as $D\approx 0.98Ca_{E}^{\prime }$. It is apparent that the experimental data collapse well into the proposed $Ca_{E}^{\prime }$. The inset of Figure 4b shows D as a function of $E^{2}$. Basically, there are two deformation regimes. When $E^{2}<{E_{c}}^{2}=3.114\times 10^{12}\;(V/m{)}^{2}$, droplet deformation has a negative value due to hydrodynamic forces. We name this regime the hydrodynamic regime (HDR), where other forces are more dominant than the electric forces. When $E^{2}>{E_{c}}^{2}$, droplet deformations are positive in value and start to deform into a prolate shape. We name this the electrical force regime (EFR). Droplet deformation resulting from the competition between hydrodynamic forces can be taken into account using the proposed $Ca_{E}^{\prime }$. When the electric field is applied, electric forces will eventually overcome the hydrodynamic forces. When the two forces are balanced out and comparable in magnitude, this results in a circular droplet or D=0.

## 5. Conclusions

In summary, we have both experimentally and theoretically studied the deformation of droplets induced by AC electric fields in a microchannel. The effects of the AC electric field strength, applied frequencies, electrical conductivities and interfacial tensions were systematically studied. Experimental results show that the droplet deformation increases linearly with increasing $aE^{2}\phi_{S}$. This is consistent with the EHD theory proposed for a static droplet in an AC electric field. The AC frequency dependence of droplet deformation at higher electric field strength is attributed to the frequency dependence of the electrical conductivity. Droplets with lower electrical conductivity deform less with increasing AC frequencies. For droplets with higher electrical conductivity, we found no dependence of deformation on the AC frequencies. In these cases, the deformation is observed to be greater than that of DI water.

The experimental finding of the electrical conductivity dependence of the droplet deformation is consistent with the theoretical calculations using the EHD theory. Our experiments and the theoretical analysis also indicates that a critical electric field strength can be used to separate the deformation into two regimes. We categorized the results using a hydrodynamic-force-dominated regime and an electric-force-dominated regime.

In the hydrodynamic force regime (HDR), where the electric field is not the dominating force, droplet deformation is dominated by the hydrodynamic forces. This accounts for the initial oblate deformation of the aqueous droplets. In the electrical force regime (EFR), the electric field dominates the hydrodynamic forces. As a result, the aqueous droplet becomes prolate. We propose a modified electric capillary number $Ca_{E}^{\prime }$ to account for the above differences. The proposed $Ca_{E}^{\prime }$ agrees well with the experimental data. Lastly, our work shows that the classical EHD theory proposed for a static droplet at the macroscale can also be applied to a moving droplet in a microchannel.

## Figures and Tables

**Table 1:** List of aqueous solutions with different electric conductivities. R refers to the conductivity ratio and S refers to the permittivity ratio. *RS* refers to the product of conductivity ratio and permittivity ratio.

Dispersed Phase	$\sigma_{2}$ (S/m)	$R=\sigma_{2}/\sigma_{1}$	$S=\epsilon_{1}/\epsilon_{2}$	RS
Deionized (DI) water I	$4.4\times 10^{-5}$	$O(10^{7})$	0.029	$O(10^{6})$
Deionized (DI) water II	$1.6\times 10^{-4}$	$O(10^{8})$	0.029	$O(10^{6})$
0.001 wt% NaCl solution	$2.7\times 10^{-3}$	$O(10^{9})$	0.029	$O(10^{7})$
0.020 wt% NaCl solution	$3.7\times 10^{-2}$	$O(10^{10})$	0.029	$O(10^{9})$
0.110 wt% NaCl solution	$2.0\times 10^{-1}$	$O(10^{11})$	0.029	$O(10^{9})$
1.010 wt% NaCl solution	$1.8\times 10^{0}$	$O(10^{12})$	0.029	$O(10^{10})$
14.774 wt% NaCl solution	$1.9\times 10^{1}$	$O(10^{13})$	0.042	$O(10^{11})$

**Table 2:** Comparison between the slope ratio $k_{e}$ from the literature and the present study. The number in parentheses reflects the nominal viscosity of the fluids.

Cases	DP	CP	$k_{e}/k_{th}$ from Visika & Saville [3]	$k_{e}/k_{th}$ from Torza et al. [2]
DC	Water	Castor oil	1.34	2.1
	Water	Silicone oil (300P)	1.26	3.8
	Water	Silicone oil (1000P)	1.4	3.8
AC (0~400 Hz)	Water	Castor oil	1	2.1
Present study AC (10~70 kHz)				
	Water	Mineral oil (30P)	$k_{e}/k_{th}=1.79$	

## Data Availability

The original contributions presented in this study are included in the article. Further inquiries can be directed to the corresponding author(s).
